# Resurgence of Medically Attended Kava-Related Exposures: An Observational Study, 2000-2024

**DOI:** 10.1192/j.eurpsy.2026.10210

**Published:** 2026-07-31

**Authors:** E. B. Towers, I. L. Williams, C. P. Holstege, R. Farah

**Affiliations:** 1 Medical Scientist Training Program; 2 Department of Emergency Medicine, Division of Medical Toxicology, University of Virginia, Charlottesville, United States

## Abstract

**Introduction:**

Kava (*Piper methysticum*), traditionally consumed in Pacific Island ceremonies, is now widely marketed as an alcohol alternative in concentrated extracts, capsules, and ready-to-drink beverages. These products are increasingly sold in vape shops near college campuses and often combined with other psychoactive substances such as kratom. This emerging trend raises significant safety concerns, yet recent patterns of kava-related toxicity remain poorly characterized, underscoring the need for ongoing surveillance.

**Objectives:**

To characterize recent trends in medically attended kava-related exposures in the United States (U.S.), including changes in demographics, clinical severity, and patterns of substance co-use.

**Methods:**

A retrospective analysis of kava-related exposures was conducted using data from the U.S. National Poison Data System between January 1^st^, 2000 and July 31^st^, 2025. Analyses assessed temporal trends, demographic shifts, clinical outcomes, and substances reported in combination with kava.

**Results:**

Medically attended kava-related exposures declined sharply in the early 2000s, from 296 in 2000 to 82 in 2004, reaching a low of 42 in 2010 (Figure 1A). Since 2016, exposures have risen steadily, reaching 180 in 2024. Both waves predominantly involved college-aged and young adults (20–39 years), but demographics shifted: the first wave was mostly women, whereas recent exposures primarily involve men (Figure 1B). Hospitalizations remained relatively stable (14–29%; Figure 2A). However, the proportion of exposures with serious clinical outcomes has more than doubled, from about 14% to over 30%. This increase coincides with rising co-use of kratom, reported in more than one quarter of cases in 2024 and one third in the first half of 2025 (Figure 2B).

**Image 1:**
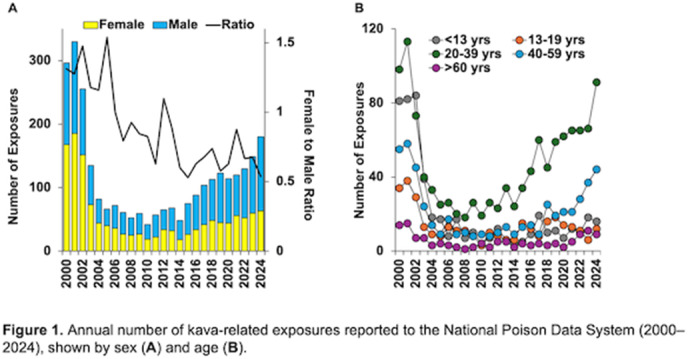
Image 1: Long description.

**Image 2:**
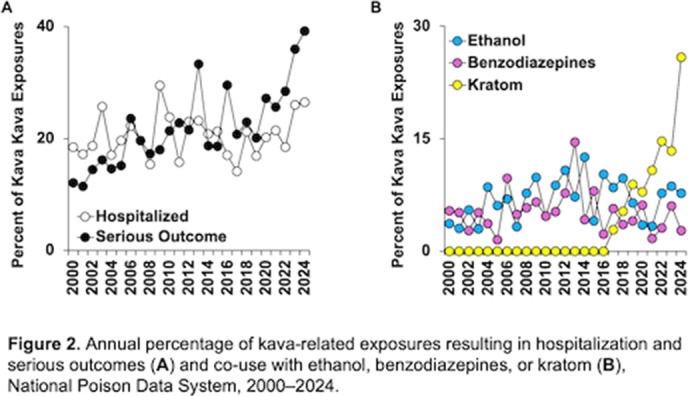
Image 2: Long description.

**Conclusions:**

The sharp decline in kava-related exposures reported to U.S. poison centers followed the 2002 U.S. Food and Drug Administration advisory warning of an association between kava use and severe liver injury. However, exposures resurged following 2016 statements suggesting low health risk with traditional use of kava. Poison centers play a critical role in early detection, and current data indicate a second wave of exposures driven by college-aged and young adults. This wave shows higher rates of serious outcomes, coinciding with rising co-use with kratom and the wide availability of products combining both substances. These trends underscore the need to evaluate safety and develop evidence-based strategies for a rapidly changing market of psychoactive plant-based products.

**Disclosure of Interest:**

None Declared

## Long descriptions

### Image 1: Long description

Panel A is a stacked bar and line graph. The x-axis represents years from 2000 to 2024. The primary y-axis on the left is Number of Exposures ranging from 0 to 300. The secondary y-axis on the right is Female to Male Ratio ranging from 0 to 1.5. Yellow bars represent Female and blue bars represent Male. A black line represents the Ratio. Total exposures peak in 2001 at over 300, decline sharply until 2010, and then show a gradual increase toward 2024. The ratio fluctuates between 0.5 and 1.5, ending near 0.5 in 2024.

Panel B is a multi-line scatter plot. The x-axis represents years from 2000 to 2024. The y-axis is Number of Exposures ranging from 0 to 120. Five age groups are tracked:

* Grey dots: less than 13 y r s.

* Orange dots: 13-19 y r s.

* Green dots: 20-39 y r s.

* Blue dots: 40-59 y r s.

* Purple dots: greater than 60 y r s.

The 20-39 y r s group shows the highest volume, peaking near 115 in 2001, dropping to a low around 2010, and rising significantly after 2015 to nearly 90 by 2024. Other age groups follow a similar U-shaped trend but at lower volumes, with the greater than 60 y r s group consistently showing the lowest number of exposures.

Navigate back to Image 1:.

### Image 2: Long description

Two-panel line graph.

Panel A. The y-axis is Percent of Kava Kava Exposures ranging from 0 to 40. The x-axis is years from 2000 to 2024. Two data series are plotted.

- Hospitalized (white circles): Fluctuates between 15 and 30 percent, ending near 25 percent in 2024.

- Serious Outcome (black circles): Shows a general upward trend, starting near 12 percent in 2000 and rising sharply after 2018 to reach nearly 40 percent by 2024.

Panel B. The y-axis is Percent of Kava Kava Exposures ranging from 0 to 30. The x-axis is years from 2000 to 2024. Three data series are plotted.

- Ethanol (blue circles): Fluctuates between 5 and 15 percent throughout the period.

- Benzodiazepines (pink circles): Fluctuates between 2 and 15 percent, showing a slight decline after 2014.

- Kratom (yellow circles): Remains at 0 percent from 2000 until 2017, then shows a rapid exponential increase, reaching approximately 25 percent by 2024.

Navigate back to Image 2:.

